# Secular trends of low birthweight and macrosomia and related maternal factors in Beijing, China: a longitudinal trend analysis

**DOI:** 10.1186/1471-2393-14-105

**Published:** 2014-03-18

**Authors:** Xiaoyi Shan, Fangfang Chen, Wenpeng Wang, Juan Zhao, Yue Teng, Minghui Wu, Honghong Teng, Xue Zhang, Hong Qi, Xiaohong Liu, Chunying Tan, Jie Mi

**Affiliations:** 1 College of Pharmacy and Health Sciences, Drake University, Des Moines, IA, USA; 2 Department of Epidemiology, Capital Institute of Pediatrics, Beijing 100020, China; 3 Beijing Obstetrics and Gynecology Hospital, Capital Medical University, Beijing, China; 4 HAIDIAN Maternity and Child Health Care Hospital, Beijing, China

**Keywords:** Low birthweight, Macrosomia, Trends, Gestational weight gain, Gestational diabetes

## Abstract

**Background:**

Information tracking changes of birthweight is scarce in China. To examine trends of low birthweight (birthweight < 2500 g) and macrosomia (birthweight ≥ 4000 g) and potential risk factors in Beijing, hospital records from two major obstetrics and gynecology hospitals in urban districts in Beijing were analyzed.

**Methods:**

Hospital records from 1996 to 2010 were retrieved. Information of prenatal examination and birth outcomes was entered into a structured database. Live births were used for trend analysis. Information of live births in 2010 was used to identify potential risk factors.

**Results:**

A total of 63 661 live births were delivered during 1996–2010 in the study hospitals. The average birthweight increased from 3271 g in 1996 to 3 359 g in 2000 and slightly declined to 3 331 in 2010. The percentage of low birthweight fluctuated around 4.0%. No significant increase or decrease was observed. Preterm birth was the main cause of low birthweight, accounting for more than 73% of low birthweight. The average percentage of macrosomia was 7.6%. The percentages of macrosomia increased from 6.6% in 1996 to 9.5% in 2000 and declined to 7.0% in 2010. Excessive gestational weight gain and gestational diabetes were significantly associated with macrosomia.

**Conclusions:**

Continuously monitoring abnormal birthweight is needed and intervention should focus on appropriate gestational weight gain and reduction of preterm birth and gestational diabetes.

## Background

Birthweight is an important indicator of the nutritional and developmental status of a newborn infant. Body mass index (BMI) before pregnancy, gestational weight gain, gestational diabetes mellitus (GDM), mother’s age and gender were reported to be associated with macrosomia (birthweight ≥ 4000) [1], while induced abortion, maternal age older than 35 years, BMI above 28 before pregnancy, stress during pregnancy, drinking and smoking may contribute to low birthweight (birthweight < 2500 g) [2]. Studies show that both low birthweight and macrosomia are related to adverse short-term and long-term health outcomes. Low birthweight is a major cause of perinatal mortality and child morbidity. Children with low birthweight are more likely to have complications and growth retardation than those with normal birthweight [3-5]. Research has shown that low birthweight is also associated with increased hypertension, metabolic disorders and other chronic diseases later in life [6]. The health consequences of low birthweight are likely to be experienced over the full course of life [6-9]. On the other end, children with macrosomia tend to gain weight faster than those born at normal weight. Abnormal weight gain in the uterus and during infancy may have an adverse influence on health in childhood and adult life. Studies show that macrosomic infants have a higher risk of developing obesity and metabolic disorders [10,11]. Macrosomia is also associated with higher risk of certain cancers [12-15]. Therefore, tracking of abnormal birthweight and related risk factors have significant public health implications.

Few studies examined secular trends of abnormal birthweight in recent years in China. One study reported an 11-year trend of macrosomia in southeast China with the prevalence increasing from 6.0% to 7.8% from 1994 to 2005 [16]. The China Department of State proposed two 10-year progress guidelines for women and children’s health in 2001. The guidelines emphasized improvement in birth outcomes and reproductive health services. In order to develop more effective prenatal care intervention to improve both mothers’ and children’s health, a better understanding of changes in birthweigh and the risk factors of abnormal birthweight in the Chinese population is needed. However, limited information is available in regard to the birthweight trend and associated factors.

The purpose of this study is to examine trends of low birthweight and macrosomia and related factors in Beijing. China has undergone a dramatic economic and nutritional transition in the past several decades [17]. Along with changes in lifestyle and diet, the epidemiology of obesity and chronic diseases has also changed [18,19]. Changes in abnormal birthweight may contribute to the current and future burden of chronic diseases, given the potential health risks of abnormal birthweight. Findings from this study could shed light on this issue and provide useful information for public health intervention. Analysis of these live-birth hospital records could provide insights into the influence of early life development on childhood and adult health.

## Methods

Two hospitals in Beijing were selected based on the availability and completeness of hospital records: Beijing Obstetrics and Gynecology Hospital (BOGH) and Haidian Maternal and Child Health Care Hospital. Both hospitals focus on services in obstetrics and gynecology and have a large volume of birth delivery services. Their hospital records are more complete and reliable than other general hospitals. A total of 63 661 live birth records were retrieved in 1996, 1997, 1998, 1999, 2000, 2005, and 2010 from the two selected hospitals. Years of information were chosen based on how useful and complete the hospital records were. Eight records were discarded when using the gender variable because of missing information. Pre-pregnancy weight and height was not collected in the records before 2000. Records after 2000 collected weight and height based on self-reported information when mothers had their first prenatal care visit (within 14 weeks). Information of 11 006 live births from BOGH in 2010 was used to examine factors related to birthweight, because records from BOGH in 2010 had complete maternal information for the analysis of related factors. Information on 9674 first-born singletons was used in the final analysis.

The study was approved by the research ethics committee of the Capital Institute of Pediatrics.

### Classifications

According to the World Health Organization [20], a low birthweight infant is one born with birthweight < 2500 g; an infant with high birthweight or macrosomia is one weighting ≥ 4000 g. Birthweight ≥ 2500 g and < 4000 g was classified as normal in the study. Gestational age less than 37 weeks was defined as preterm and between 37–41 weeks as normal term. Body mass index (BMI) was calculated from self-reported height and weight before pregnancy (BMI = weight in kg/height^2^ in meter). According to BMI cutoffs for overweight and obesity in Chinese [21], maternal prepregnancy weight was classified into underweight (BMI < 18.5), normal weight (18.5 ≤ BMI < 24), overweight (24 ≤ BMI <28) and obesity (BMI ≥28).

The adequacy of gestational weight gain during pregnancy was classified into inadequate, adequate and excessive using the new recommendations from the Institute of Medicine (IOM) [22]. These recommendations were based on maternal prepregnancy BMI. The criteria used in this study were consistent with the recommendations according to weight status classification.

Pregnancy-induced hypertension (PIH) in this study was defined as hypertension (systolic blood pressure ≥ 140 mHg and/or diastolic blood pressure ≥ 90 mHg) that developed after 20 weeks with or without proteinuria. According to the recommendation from the Chinese Medical Association Obstetrics Group [23], GDM was diagnosed when one of the following condition was met: 1) fasting plasma glucose ≥ 5.8 mmol /L twice or more times; 2) two or more test results are equal or above the following values after a 100 g-load oral glucose tolerance test: fasting, 5.8 mmol/L; 1-hour, 10.6 mmol/L; 2-hour, 9.2 mmol/L; 3-hour, 8.6 mmol/L; 3) 50 g glucose challenge test ≥ 11.1 mmol/L and fasting plasma glucose ≥ 5.8 mmol/L. Since diagnosis criteria have changed overtime in the past decades, data about PIH and GDM was only collected from the BOGH in 2010 to ensure the consistency of the data. The diagnosis of PIH and GDM was confirmed by obstetricians.

### Statistical analysis

All information was entered in to EpiData3.1. One-way ANOVA was conducted to compare the means of birthweight among groups. Linear regression was performed to examine linear trends in birthweight over time using birthweight as the dependent variable. Percentages of low birthweight and macrosomia were compared using *chi* square analysis for trends. Data in 2010 were analyzed to identify risk factors for abnormal birthweight. Multivariable logistic regression models were used to examine the association between maternal characteristics and abnormal birthweight and potential risks. Stepwise logistic regression was used for modeling to select independent variables among mother’s age, pre-pregnancy BMI, adequacy of gestational weight gain, amniotic fluid volume, gestation, gestational diabetes, PIH, newborns’ gender, mother’s education and nationalities. These variables were considered based on literature review for their relevance to abnormal birth weight and availability in the sample. Independent variables were selected by forward selection using maximum likelihood estimates and a significance level of 0.05. In logistic regression analysis only first-born singletons’ data was used since most children were first-born singletons under the one-child policy during the study period. Consistency and plausibility checks were conducted after data entry to minimize errors. Data analyses were performed with SPSS18.0 (SPSS, Chicago). The statistical significance was inferred at a 2-tailed P < 0.05.

## Results

### Maternal and infant characteristics

Maternal and birth outcome information is shown in Table 1. Birthweight increased from 1996 to 2000 and slightly declined afterward. Linear regression analysis shows there was an increase overall in the period of 15 years (P < 0.001). The increase before 2000 and the decrease after 2000 were also statistically significant. Further analysis shows average birthweight between years before and after 2000 was significantly different (before 2000, *P* < 0.001; after 2000, *P* < 0.001). Among a total of 63661 live births delivered during 1996–2010, 33402 (52.5%) were boys and the ratio of boys to girls was 110:100. No significant change in the ratio of gender was observed in the past 15 years. There was a steady increase in preterm birth from 1999 to 2010. The average birthweight is 3329 g (95% CI:2250-4250 g). Birthweight of male infants was about 4% higher than that of female infants.

**Table 1:** Maternal and neonatal characteristics of live births in Beijing, 1996-2010

**Years**	**1996**	**1997**	**1998**	**1999**	**2000**	**2005**	**2010**	**1996-2010**	**1996-2000**	**2000-2010**
**Infants**								**P**	**P**	**P**
Live births (*n*)	3498	3987	3750	4278	8528	17105	22507	-	-	-
Boys % (n)	52.7(1842)	52.8(2105)	51.9(1947)	51.5(2202)	53.0(4520)	52.5(8983)	52.4(11803)	0.95^a^	0.86 ^a^	0.43 ^a^
Mean ± SD birthweight g	3271 ± 475	3281 ± 480	3316 ± 473	3336 ± 477	3359 ± 499	3337 ± 502	3331 ± 488	<0.001^b^	<0.001 ^b^	<0.001 ^b^
Boys g	3327 ± 479	3340 ± 473	3368 ± 469	3397 ± 478	3412 ± 507	3392 ± 514	3383 ± 490	<0.001 ^b^	<0.001 ^b^	<0.05 ^b^
Girls g	3209 ± 463	3217 ± 473	3259 ± 472	3272 ± 468	3300 ± 483	3277 ± 482	3275 ± 481	<0.001 ^b^	<0.001 ^b^	<0.05 ^b^
Birthweight < 1500 g % (*n*)	0.2(8)	0.2(6)	0.2(8)	0.3(14)	0.3(29)	0.5(92)	0.5(115)	<0.001 ^a^	0.06 ^a^	0.12 ^a^
Birthweight < 2500 g % (*n*)	4.3(151)	4.1(164)	3.3(123)	3.5(148)	3.8(324)	4.9(845)	4.6(1045)	<0.001 ^a^	0.16 ^a^	0.05 ^a^
Birthweight ≥ 4000 g %(*n*)	6.6(231)	6.6(263)	7.7(288)	7.9(340)	9.5(808)	8.0(1360)	7.0(1579)	0.85 ^a^	<0.001 ^a^	<0.001 ^a^
Birthweight ≥ 4500 g %(*n*)	0.7(23)	0.8(31)	1.1(42)	1.1(47)	1.2(106)	0.8(143)	0.6(146)	<0.05 ^a^	<0.05 ^a^	<0.001 ^a^
Gestation < 37 wk %(*n*)	4.1(142)	5.0(200)	4.4(165)	4.2(180)	5.9(507)	6.5(1119)	6.6(1496)	<0.001 ^a^	<0.001 ^a^	<0.05 ^a^
Multiple live births %(*n*)	2.1(72)	2.3(91)	1.8(68)	2.3(97)	2.4(203)	2.7(460)	2.6(587)	0.001 ^a^	0.24 ^a^	0.39 ^a^
**Mothers**										
Mean age yrs	26.5	26.7	27.0	27.3	28.1	29.4	29.9	<0.001 ^b^	<0.001 ^b^	<0.001 ^b^
Primiparity %(*n*)	90.0(3115)	91.2(3595)	88.6(3294)	89.0(3765)	90.3(7604)	86.7(14530)	89.7(19900)	<0.05 ^a^	0.72 ^a^	0.07 ^a^
Age % (*n*)										
< 20 yrs	0.4(15)	0.5(19)	0.5(17)	0.6(24)	0.3(21)	0.2(28)	0.2(54)	<0.001 ^a^	0.07 ^a^	0.70 ^a^
20-34 yrs	97.5(3357)	97.3(3811)	96.5(3567)	96.4(4052)	94.1(7896)	89.2(14989)	88.3(19522)	<0.001 ^a^	<0.001 ^a^	<0.001 ^a^
≥ 35 yrs	2.0(70)	2.2(86)	3.0(111)	3.0(126)	5.6(474)	10.6(1782)	11.5(2532)	<0.001 ^a^	<0.001 ^a^	<0.001 ^a^
Prepregnancy BMI kg/m^2^, mean ± SD ^c^	-	-	-	-	21.3 ± 3.0	21.1 ± 2.9	21.0 ± 3.0	-	-	<0.001 ^b^
Prepregnancy BMI kg/m^2^, % ^c^ (*n*)										
< 18.5	-	-	-	-	14.7(451)	16.8(1220)	17.9(1859)	-	-	<0.001 ^a^
18.5-24	-	-	-	-	68.5(2106)	69.3(5032)	68.2(7070)	-	-	0.39 ^a^
≥ 24	-	-	-	-	16.8(517)	13.8(1005)	13.9(1442)	-	-	<0.05 ^a^
Gestational weight gain kg, mean ± SD^c^ (*n*)	-	-	-	-	16.3 ± 5.7	17.0 ± 5.4	16.8 ± 5.1	-	-	<0.001 ^b^
Inadequate^d^ %(*n*)					16.2(460)	12.6(883)	11.7(1206)	-	-	<0.001 ^a^
Adequate^d^ %(*n*)					30.3(862)	28.2(1973)	31.9(3277)	-	-	<0.05 ^a^
Excessive^d^ %(*n*)					53.5(1524)	59.1(4130)	56.4(5802)	-	-	0.34 ^a^
PIH^c^ (%) (*n*)							4.7(509)	-	-	-
GDM^c^ % (*n*)							4.0(431)	-	-	-
Education^c^ %(*n*)										
Elementary school or less)	-	-	-	-	-	1.3(99)	0.3(18)	-	-	-
Middle or high school	-	-	-	-	-	47.4(3668)	11.7(753)	-	-	-
College or more	-	-	-	-	-	51.3(3967)	88.0(5650)	-	-	-

BMI, body mass index, SD, standard deviation, PIH, pregnancy-induced hypertension, GDM, gestational diabetes mellitus.

^a^Chi square tests for trend were used to compute P values.

^b^Linear regression was used to compute P values.

^c^Data source: Beijing Obstetrics and Gynecology Hospital; the total number of live births used in the analysis was 10802.

^d^Based on recommendations from the Institute of Medicine.

PIH is defined as hypertension (systolic blood pressure ≥ 140 mHg and/or diastolic blood pressure ≥ 90 mHg) that developed after 20 weeks with or without proteinuria. GDM was diagnosed when two or more of the plasma glucose threshold levels were met after a 100 g-load oral glucose tolerance test: fasting, 5.3 mmol/dL; 1-hour, 10.0 mmol/dL; 2-hour, 8.6 mmol/dL; 3-hour, 7.8 mg/dL.

The maternal age for giving birth increased significantly. The mean age of giving birth was 26.5 years in 1996 and increased to 29.9 years in 2010. The proportion of women older than 30 years increased significantly. Especially, the proportion of women 35 years or older in 2010 (11.5%) was fivefold of that in 1996 (2.1%). No significant change in maternal weight status was observed, though this information was only available after 2000. The gestational weight gain stayed at a relatively high level from 2000 onwards compared to IOM recommendations. The average weight gain in normal weight, overweight and obese women amounted to 1.1, 3.5 and 4.0 kg respectively, which corresponds to the upper level of IOM recommendations.

### Change in the prevalence of abnormal birthweight

**Figure 1:**
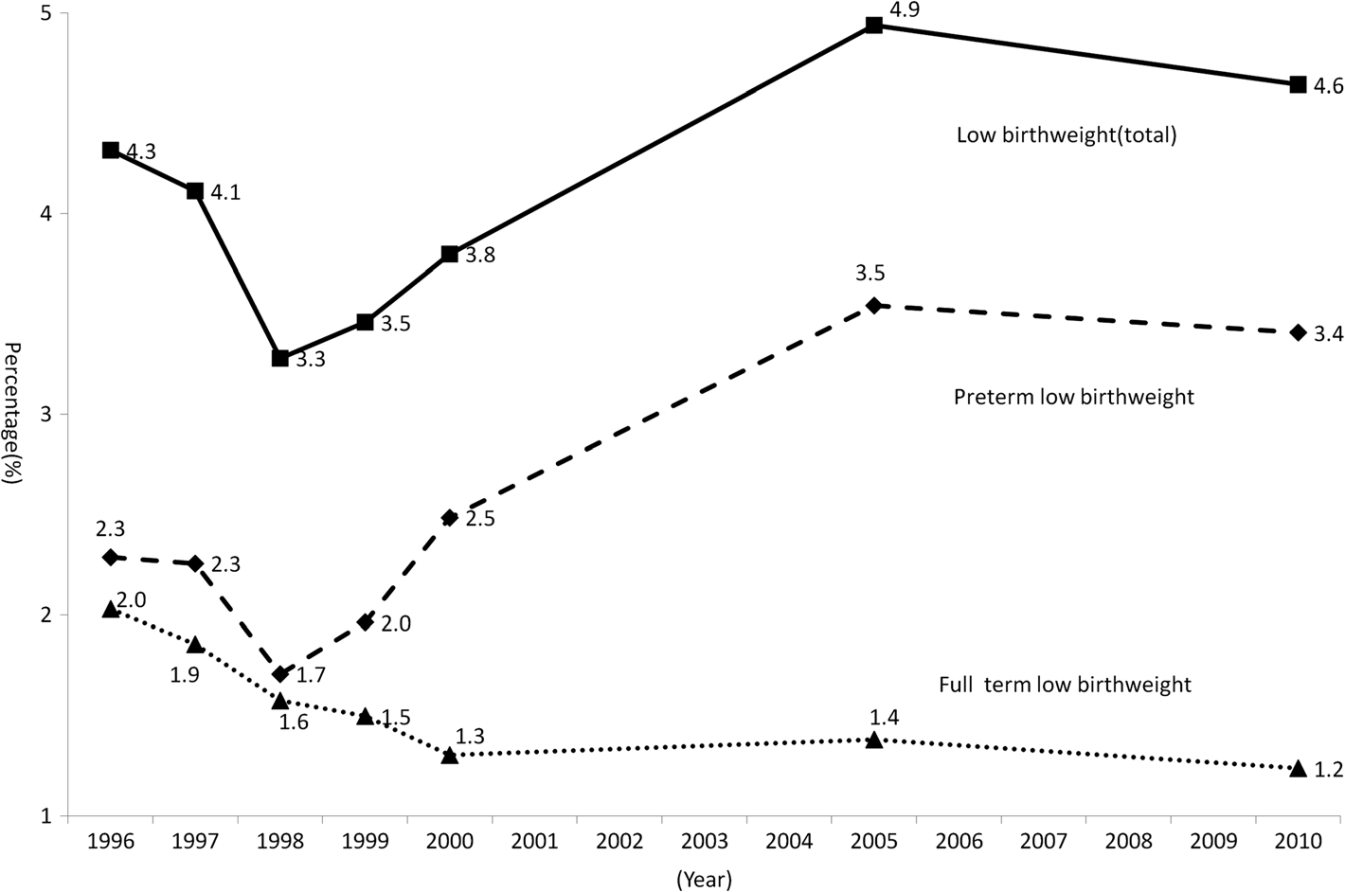
Percentage of full term low birthweight and preterm low birthweight from 1996 to 2010.

There is no clear trend of change in low birthweight percentages during this period of time (Figure 1) (*P* > 0.05), though an increase in very low birthweight percentage (birthweight < 1500 g) was observed (data shown in Table 1). The low birthweight percentages varied around 4.0%. The percentage of preterm low birthweight infants seemed to increase over time (*P* = 0.017). Further analysis shows the portion of preterm low birthweight increased markedly over time. Among low birthweight infants, 73% were preterm in 2010. The figure also shows that the percentage of low birthweight infants born full term declined from 2.0% in 1996 to 1.2% in 2010.

The percentage of macrosomia increased by the year of 2000 and slightly declined in 2005 and 2010 (Table 1). The average percentage of macrosomia was 7.6% in the past 15 years. The change in birthweight ≥ 4500 g showed a similar pattern.

### Factors related to abnormal birthweight

When computing adjusted odds ratios for non-normal birthweight, stepwise logitstic regression using forward selection was performed to build the final models (Table 2). The results show increased odds of low birthweight to be related to inadequate gestational weight gain, oligohydramnios, preterm birth, PIH and baby’s gender. Macrosomia was associated with overweight/obesity, excessive gestational weight gain, polyhydramnios, gestation more than 40 weeks and gestational diabetes and baby’s gender.

**Table 2:** **Logistic regression on factors related to low birth weight and macrosomia**^**a**^

**Factors**^**b**^		***OR *** **[95 % CI]**
**Low birth weight**		
Gestational weight gain	Inadequate	2.38[1.39-4.08]
	Adequate	1
Gestation	<37 weeks	80.88[54.23-120.61]
	37 weeks or more	1
PIH	Yes	2.40[1.25-4.61]
	No	1
Amniotic fluid volume	Oligohydramnios	3.14[1.57-6.29]
	Normal	1
Baby gender	Male	1.66[1.12-2.46]
	Female	1
**Macrosomia**		
Gestational weight gain	Excessive	2.46[1.85-3.27]
	Adequate	1
Gestation	40 weeks or more	2.94[2.36-3.66]
	37-40 weeks	1
Gestational diabetes	Yes	1.89[1.16-3.09]
	No	1
Amniotic fluid volume	Polyhydramnios	3.00[1.50-6.04]
	Normal	1
Baby gender	Male	1.77[1.42-2.22]
	Female	1
Prepregnancy BMI	Underweight	0.46[0.31-0.69]
	Normal	1
	Overweight/obesity	1.99[1.53-2.59]

OR, odds ratio, CI, confidence interval, BMI, body mass index, PIH, pregnancy-induced hypertension.

^a^Data source: Beijing Obstetrics and Gynecology Hospital, 2010.

^b^Factors were selected using stepwise regression forward selection at a significance level of 0.05.

Pregnancy-induced hypertension and gestational diabetes diagnosis were defined in Table 1.

Since a considerable number of women had excessive gestational weight gain, which is one of the risk factor for abnormal birthweight, the percentage of meeting or not meeting the IOM recommendations was tabulated according to their BMI categories (Table 3). No more than 30% of women with BMI between 18.5-25 met the recommended gestational weight gain. Regardless of weight status, women tended to gain more weight than recommended. The heavier women were before pregnancy, the more weight they gained during pregnancy (P < 0.001).

**Table 3:** **Gestational weight gain according to BMI categories **^**a**^

	**Inadequate**^**b**^			**Adequate**^**c**^			**Excessive**^**d**^		
	**kg**	**n**	**%**	**kg**	**n**	**%**	**kg**	**n**	**%**
BMI < 18.5 kg/m^2^	<12.70	235	13.6	12.70-18.14	762	44.3	>18.14	725	42.1
BMI 18.5-24.9 kg/m^2^	<11.34	768	11.4	11.34-15.88 g	2004	29.7	>15.88	3970	58.9
BMI 25–29.9 kg/m^2^	<6.80	50	7.1	6.80-11.34 g	156	22.1	>11.34	499	70.8
BMI ≥ 30 kg/m^2^	<4.99	4	3.3	4.99-9.07	24	19.5	>9.07	95	77.2
Total		1057	11.4		2946	31.7		5289	56.9
Linear trend analysis									
P ^e^			<0.001			<0.001			<0.001

BMI, body mass index

^a^Data source: Beijing Obstetrics and Gynecology Hospital, 2010.

^b^Weight gain less than recommended by the Institution of Medicine (IOM).

^c^Weight gain in line with IOM recommendations.

^d^Weight gain higher than recommended by IOM.

^e^P values come from chi square for trend analysis.

## Discussion

This study is the latest one that has examined the abnormal birthweight trends and related factors in the region. There has been no significant change in average birthweight in the past 15 years. It increased until 2000, and decreased since then. A woman’s age at the time of having a first birth increased significantly. The proportion of low birth weight varied between 3.3% and 4.9%, with no clear change patterns. The proportion of macrosomia increased from 6.6% in 1996 to 9.5% in 2000, then decreased to 7.0% in 2010.

We believe our results represent the change of birthweight in the Beijing urban area for the past 15 years. Women who chose the two selected hospitals were regular residents of Beijing. They were presumably not different in demographics from others. Births in our study hospitals in 2005 and 2010 accounted for 17.2% and 14.0% of all births in Beijing, respectively. Hospital births accounted for 99.2% of all births in cities in 2010 [24]. Therefore, hospital records of live births represent all births in the catchment areas reasonably well.

The percentage of low birthweight in the study was higher than that in other major cities. Data show the percentage of low birthweight was 4.6% at the national level, 3.9% in cities and 4.8% in rural areas in 2005 [25]. The percentage of low birthweight was 4.9% in a 2005 study based on medical obstetric records from the two obstetrics and gynecology hospitals in Beijing. It is not surprising to see a higher percentage of low birthweight in live births in Beijing, because with more advanced medical care, survival rates of low birthweight and preterm newborns are likely higher in Beijing than the national average. However, the percentage is relatively low in the international comparison. A report from the United Nations Children’s Fund showed the percentage of low birthweight was 14% globally during 1998–2002 [26]. Data from several developed counties show the percentage was 8%, 7%, 6%, and 8% during 2005–2009 for the United States, Australia, Canada, and Japan respectively [27].

It appears that there has been no significant reduction in percentages of low birthweight and preterm births in the past 15 years. The increase in preterm live births was observed in recent years. This could be due to the advanced perinatal care which has increased the survival rate of high-risk births including extremely preterm births and births with extremely low birthweigth. A national survey showed infants with birthweight less than 1500 g accounted for 3.8% of low birthweight in 1998 in major cities [28], while the proportion of birthweight less than 1500 g was 10.9% in this study. The infants born full term with low birthweight were only 1.2% of the total live births in 2010. Among low birthweight, our data show 73% of the low birthweight infants were preterm births. As mentioned above, with increased survival rates of preterm births and extremely low birthweight newborns, it is logical that preterm birth becomes the primary cause of low birthweight in this region. Further exploration on underlying causes of preterm birth is needed to fully understand the risk factor of low birthweight in the population.

Pregnancy-induced hypertension was also found to be positively associated to low birthweight in this study. This finding is consistent with several other studies [29,30]. In a Malaysian population, women with pregnancy-induced hypertension were four times more likely to have low birthweight babies [30]. As many studies show, individuals with low birthweight, in turn, were found to have increased risk of metabolic disorders and cardiovascular diseases. In addition, PIH and preeclampsia are an independent risk factor of elevated blood pressure in offspring during both childhood and adulthood [31,32]. Considering the potential adverse health consequences in offspring, PIH should be the target of intervention in this population.

The prevalence of macrosomia was relatively high varying from 6.6% to 9.5%, and the secular trend was similar to a recent report of macrosomia in southeastern cities in China [16], experiencing an increase from 1996 to 2000 and then a decline from 2005 to 2010. Increased birth size and macrosomia have been observed in many developed counties. Reports from Norway, Sweden and Canada using national data indicate a yearly increase in birthweight of approximately 3 g from about 1970 to 2000 [33,34]. Birthweight is positively associated with childhood and adulthood BMI [35-37]. Infants born with macrosomia may not only be prone to obstetric complications and neonatal mortality, but may also be at greater risk of obesity and other features of chronic disease later in life. Cardiovascular diseases are already the leading cause of mortality and a major public health concern in China [19]. Infants with macrosomia could generate another pool of cardiovascular disease patients which add the already heavy disease burden. The percentage of macrosomia peaked around 2000 and declined afterwards. This may be due to increased awareness and a concerted effort that begun in 2001 to educate pregnant women and help them gain weight during pregnancy appropriately.

Of note, gestational weight gain exceeding the recommended amount is a concern because it is strongly related to macrosomia in the study. More than half of the women in the study gained more weight than recommended during pregnancy, while inadequate weight gain was of less concern. Further analysis shows that heavier women were more likely to exceed the recommended weight gain during pregnancy. These findings may reflect the traditional perception of weight gain during pregnancy—‘the bigger and heavier, the better’. This traditional notion came from the time when food was scarce and malnutrition was prevalent both among children and pregnant women in China. With the economic growth and great improvement in nutrition and living conditions during the past several decades, food has become more readily available than ever before. Dietary structure in the Chinese population has changed towards high calories, high fat and low fiber [38]. Over-nutrition issues (or another form of malnutrition) have become more evident in many major cities and urban areas in China. Studies have shown that excessive weight in children, pregnant women and adults, has a profound health impact. In addition, IOM recommendations on gestational weight gain, which are based on western populations, have been used as guidelines by Chinese medical professionals. Appropriate guidelines for Chinese women need to be discussed and developed in consideration of differences in body composition, diet and cultural practices between Western populations and Asians.

This study was able to update the percentages of abnormal birthweight by utilizing 15-year obstetric records from two large obstetrics and gynecology hospitals in Beijing. However, some limitations need to be mentioned. Though the obstetric records provided relatively complete information about birth outcomes and maternal characteristics, we were not able to assess individual lifestyle factors such as diet, smoking and drinking that are reported to be related to birth outcomes. Due to limited resources, we were not able to assess data annually during 2000–2010, rather we included data of every five years to represent the change during this period of time. In addition, the results in the study only represent metropolitan and urban areas. Rural areas may have different patterns in the change of abnormal birthweight. Further study should be conducted to assess the percentages of low birthweight and macrosomia in non-urban regions in order to make appropriate recommendations and interventions. The number of available hospital records increased dramatically after 2000 because of improved completeness of records, an increase of the population, particularly of migrant workers [39], and the expansion of the obstetrics and gynecology hospitals. Though we were not able to compare present with absent records before 2000, we believe that any potential bias is likely to be non-directional, and may lead to a loss of precision but not of validity.

## Conclusions

While the prevalence of low birthweight was maintained at a relatively low level, the prevalence of macrosomia was high over the past 15 years, which might have contributed to the current sharp rise in overweight and obesity among children and adolescents. As many studies show, birthweight is associated with health outcomes. Therefore, low birthweight and macrosomia should be closely monitored in order to plan prompt and effective intervention. Continuing efforts should be taken to address preterm birth, gestational weight gain as well as PIH and gestational diabetes, all of which may contribute to abnormal birthweight.

## Competing interests

The authors declare that they have no competing interests.

## Authors’ contributions

XS, FC and WW contributed to the data analysis and manuscript writing. HT, JZ, YT, MW, XZ, HQ, XL, CT and JM contributed to the data collection, analysis and interpretation of the results. JM conceived, designed and directed this study, made critical revisions of the manuscript. All authors read and approved the final manuscript.

## Pre-publication history

The pre-publication history for this paper can be accessed here:

http://www.biomedcentral.com/1471-2393/14/105/prepub
